# On Cold Bathing in Rheumatism

**Published:** 1803-06-01

**Authors:** 


### *To the Editors of the Medical and Physical Journal.*

Gentlemen,

I Conceive it might be admitted as an axiom in philosophy, that knowledge, unless it be scientific and founded upon principles, ought to be considered as a suspicious light which may mislead. It may be said of ———,

> No system guides th' unscientific man,

> All is a chaos or a random plan.

Science, I believe, is defined a particular kind or branch of knowledge, wrought by theory and demonstration to clear, deducible, and self-evident principles. We traverse the regions of knowledge, we walk in the paths of science. These metaphors I consider to be descriptive of the sense, and, like all good metaphors, the meaning is not difficult to discover: one implies a wandering, vague, and without controul; the other a definitive and circumscribed action. The promulgation of experience is therefore of great importance to the cause of science; since it is chiefly from the combination of facts, that science was and can be formed. Your Medical Journal appears to me to possess this genuine principle of philosophy. Its fundamental tenets are, a detail of medical facts, faithfully composed from the unquestionable experience of divers medical practitioners. The case I have to offer you is sterling; it is of my own person.

Nearly two years ago, after a day of great fatigue, I had occasion to walk, *au soir*, over a considerable extent of pasturage land, the grass of which was wet. I had not proceeded far before I began to feel uneasiness in my right ancle and foot, which before the end of my ambulation became so very painful, that I was unable to walk without frequent haltings. I obtained no remedy from rest, for the next day the complaint appeared to be aggravated. The foot was swoln and turned awry, bending inwardly; this deformity was so great, that a medical acquaintance affirmed there must be a luxation of bone. I knew however the complaint was only muscular. The ancle and foot were coloured with a rheumatic redness. In a few weeks erratic rheumatism afflicted every joint of my body. I had sometimes lumbago, at other times rheumatism in my shoulders, so badly that it was with difficulty I could put on my coat; in my wrist, that I could not supinate my hand; in my knuckles, that I could not pare an apple. In truth, for upwards of a year I was lame and decrepit.

— To

—To be plain, my infirmities began at last to acquire me the odious appellation, of "The lame Doctor." You may justly suppose I was very solicitious to get rid of my vexatious companion, Rheumatism. I used most of the medicines commonly prescribed for that disease, and also electricitry. As for electricity, I should have been equally benefited had I merely rotated the electrical wheel. It is worth remarking, that soon after the accession of rheumatism, I removed to another part of the globe. I dwelled four months within the tropic of Cancer; yet a hot climate, and all the medicines I had taken, did not procure me a remission of the disease. Returning to England in May last, I determined to try the effect of immersions into cold spring water. I continued indefatigably the use of the immersions for nearly four months. I constantly bathed once, frequently twice, and sometimes three times a day. At each immersion I usually swam about in the water for a few minutes. For the first month the bathing seemed to have no other effect upon me than a remission of rheumatism while I was in the water. In the course of the second month I was more encouraged to proceed in my cold plan. At last I relinquished the bathing, because it was unnecessary to seek further for what I had already found, *a perfect recovery.* It may be proper to observe, that during the bathing I adopted the use of fleecy hosiery stockings, which I believe materially assisted in the completion of the cure. I shall be cautious of betraying the Sciolist, shall assume no principle, offer no inference, nor involve confidence with this case, which in my experience is a solitary fact. The case was such as I have here related. If you think it will add to the stock of useful information, you are welcome to print this letter. If you see any errors in my letter, by all means correct them. I am apt to think, had such an injunction been given you by *some* of your authors, we should not see the Medical Journal so disfavoured as it is *sometimes* in point of style and language. I say nothing of the refinement of letters, of sublimity in writing, and the like, because among the gay flowers of rhetoric, an aukward metaphor and an ill chosen simile, are not unfrequently the weeds of the best cultivated soil.

I am, &c.

*April* 8, 1802. S. G.

